# One immune cell to bind them all: platelet contribution to neurodegenerative disease

**DOI:** 10.1186/s13024-024-00754-4

**Published:** 2024-09-27

**Authors:** Gabriela Rodriguez Moore, Isabel Melo-Escobar, David Stegner, Oliver Bracko

**Affiliations:** 1 Department of Biology, University of Miami, Coral Gables, FL 33146 USA; 2 Neuroscience Program, University of Miami Leonard M. Miller School of Medicine, Miami, FL 33136 USA; 3 Institute for Experimental Biomedicine, University Hospital Würzburg, Würzburg, Germany; 4 Rudolf Virchow Center for Integrative and Translational Bioimaging, Julius-Maximilians University of Würzburg, Würzburg, Germany; 5 Department of Neurology, University of Miami Leonard M. Miller School of Medicine, Miami, FL 33136 USA

## Abstract

Alzheimer’s disease (AD) and related dementias (ADRD) collectively affect a significant portion of the aging population worldwide. The pathological progression of AD involves not only the classical hallmarks of amyloid beta (Aβ) plaque buildup and neurofibrillary tangle development but also the effects of vasculature and chronic inflammatory processes. Recently, platelets have emerged as central players in systemic and neuroinflammation. Studies have shown that patients with altered platelet receptor expression exhibit accelerated cognitive decline independent of traditional risk factors. Additionally, platelets from AD patients exhibit heightened unstimulated activation compared to control groups. Platelet granules contain crucial AD-related proteins like tau and amyloid precursor protein (APP). Dysregulation of platelet exocytosis contributes to disease phenotypes characterized by increased bleeding, stroke, and cognitive decline risk. Recent studies have indicated that these effects are not associated with the quantity of platelets present in circulation. This underscores the hypothesis that disruptions in platelet-mediated inflammation and healing processes may play a crucial role in the development of ADRD. A thorough look at platelets, encompassing their receptors, secreted molecules, and diverse roles in inflammatory interactions with other cells in the circulatory system in AD and ADRD, holds promising prospects for disease management and intervention. This review discusses the pivotal roles of platelets in ADRD.

**Supplementary Information:**

The online version contains supplementary material available at 10.1186/s13024-024-00754-4.

## Introduction

It is estimated that in the United States, 1 in 47 people are affected by Alzheimer’s disease (AD) and related dementias (ADRD). Among various types of dementia, AD is the most common, followed by vascular dementia, Lewy Body Dementia, and mixed dementia, respectively [1, 2]. The progression of AD is accompanied by vascular conditions, systemic inflammation, and neuroinflammatory processes, alongside the classical pathological hallmarks of Aβ plaques and neurofibrillary tangles [3–7]. Understanding the detailed molecular, cellular, and vascular mechanisms of inflammation at play in these neurodegenerative diseases is critical to developing the innovative therapeutics necessary to aid with these conditions. Neuroinflammation in ADRD is generally believed to be triggered by cerebral cellular damage or the abnormal accumulation of pro-inflammatory molecules in the central nervous system (CNS) [3, 8]. Activation of innate and acquired immunity can contribute to increased neuroinflammation [9]. Inflammation and CNS pathologies exacerbate each other *via* diverse cellular and molecular mechanisms, such as oxidative stress or the accumulation of pro-inflammatory molecules induced in the damaged CNS tissue [10].

Platelets are metabolically active but anucleated cells produced by polyploid megakaryocytes in a process referred to as thrombopoiesis [11, 12]. Megakaryocytes release platelets into the bloodstream through pseudopodial projections, which maintain platelet counts between 150 and 450 billion per liter of blood in humans [11, 12]. Several studies have elucidated a link between immune cell involvement and the development of AD [13, 14]. Recently, platelets have received greater attention for their pivotal roles in neuroinflammation and neurodegeneration. Patients with higher baseline levels of platelet activation have a correlation with faster rates of cognitive decline, regardless of age, gender, or cardiovascular risk [15, 16]. This was characterized by increased activation of glycoprotein IIb-IIIa, the major platelet integrin adhesion receptor, and P-selectin, which serves as a marker for platelet degranulation, amongst other factors discussed later [15, 17]. Additionally, platelet A_2A_ – an adenosine receptor, monoamine oxidase-B (MAO-B) – a mitochondrial membrane enzyme that catalyzes oxidative deamination and plays a role in neuroactive and vasoactive amine metabolism, high molecular weight / low molecular weight tau (HMW/LMW tau), nitric oxide (NO), and Peroxynitrite (ONOO-) were all shown to be upregulated in AD [18–21]. While AD patients also demonstrated reduced APP ratios, A disintegrin and metalloprotease 10 (ADAM10) – a proteolytic cell surface protein whose responsibilities include cleaving APP, and Na+ -K + ATPase expression [19, 22]. A recent study on platelets from young, AD, and elderly patients identified an elevated proteomic signature, with 137 differentially expressed proteins, indicative of increased platelet activation compared to young controls. This study also showed potential dysregulation in the ubiquitin-proteasome system in AD [23]. Platelets from patients with AD displayed a higher fraction of activated platelets under steady-state conditions than those in control groups [16]. This suggests that platelets could play a crucial role in the development of AD and other forms of dementia and supports the hypothesis that platelet involvement in inflammation and healing may be altered in such diseases.

Platelets are best known for their role in primary hemostasis, where – in response to injury – they become activated, aggregate, and form clots to seal wounds. This process is closely intertwined with plasmatic coagulation, a complex process that involves the interaction of multiple bioactive mediators and cellular components [24, 25]. Of note, platelets do not only aggregate with each other or with the vessel wall, but they can also interact with various immune cells, including leukocytes and T cells in processes summarized under the term thrombo-inflammation [26–28]. Likewise, zymogens play a central role in the activation of coagulation and bridging communication between platelets and other immune cells [24, 25]. Meanwhile, major coagulation factors, such as tissue factor, fibrinogen, and thrombin, are drivers of inflammation [29–33]. Under normal physiological conditions, these intertwined systems work in homeostatic balance, but dysregulation of this crosstalk likely contributes to cellular injury and disease pathogenesis. In this review, we summarize recent evidence on platelet-centered immune responses in vascular inflammation in the context of neurodegenerative diseases.

## A well-equipped cell: platelet receptors and secreted molecules in inflammation

**Fig. 1:**
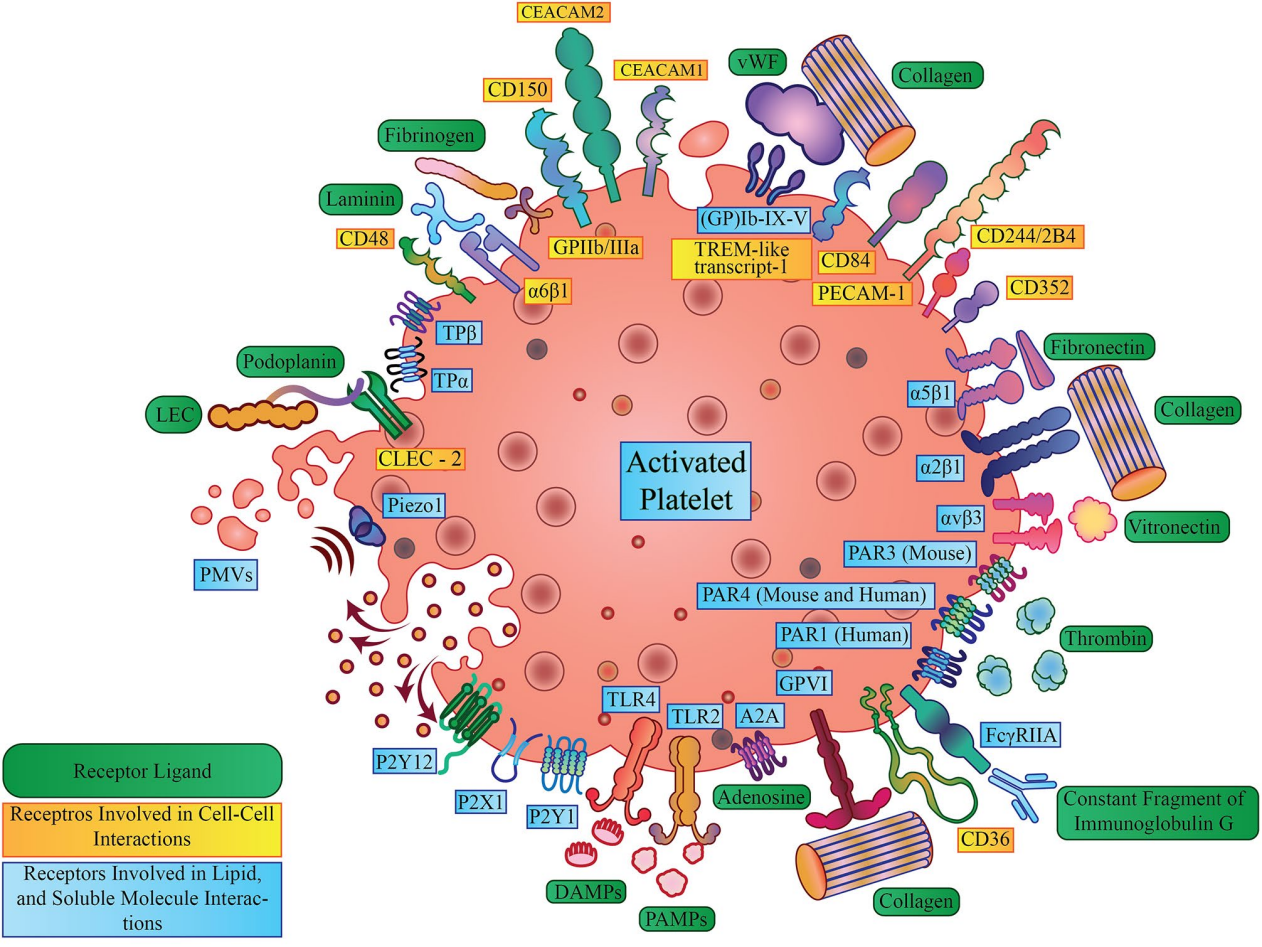
Receptors Expressed on Activated Platelet Cell Surface. The figure illustrates the main receptors expressed on the cell surface of platelets, including Toll-like receptor 2 (TLR-2), Toll-like receptor 4 (TLR-4), TREM-like transcript 1 (TLT-1), platelet glycoprotein 4 (CD36), (GP)Ib-IX-V, C-type lectin-like receptor 2 (CLEC-2, CLEC-1b), P2Y12, P2 × 1, P2Y1, Piezo 1, GPVI, PAR1, PAR3, PAR4, αvβ3, α2β1, α5β1, CD352, CD244/2B4, PECAM-1 CD84, CEACAM1, CEACAM2, (GP)IIb/IIIa, CD150, α6β1, CD48, A_2A_ Adenosine receptor, and several of their key agonists in inflammation including Damage-associated molecular pattern molecules (DAMPs), Pathogen-associated molecular patterns (PAMPs), Fibrinogen, Fibronectin, Laminin, vWF, Collagen, Constant Fragment of Immunoglobulin G, Thrombin, Vitronectin, and Podoplanin expressed by Lymphatic endothelial cells (LECs). Furthermore, the formation of PMVs and the exocytosis of soluble molecules from platelet granules are highlighted on the left-hand side of the figure

Platelets have numerous membrane-associated and soluble proteins that modulate inflammation and inflammatory diseases [28, 34]. While some of them facilitate direct platelet-immune cell interactions, others are secreted upon platelet activation from the platelets’ granules [35–38]. These give platelets a diverse arsenal of tools with which they are able to interact with the vascular environment and influence the activity of other immune cells. In the subsequent sections, we will discuss numerous key platelet receptors and secreted molecules implicated in neuroinflammation and neurodegeneration, with key ones highlighted in Fig. 1.

### Platelet receptors

The membrane glycoproteins of human platelets act as receptors that mediate adhesion to the subendothelial matrix and an array of platelet interactions [39, 40]. Initial platelet adhesion to the blood vessel wall under high-shear stress is achieved by the binding of the (GP)Ib-IX-V complex on platelet cell surface to von Willebrand factor (vWF) expressed on endothelial cells and/or bound to collagen, at sites of injury [41–43]. Deficiency of any of the complex subunits, such as (GP)Ibα and (GP)Ibβ, – with the exception of the (GP)V subunit– results in complete absence of the (GP)Ib-IX-V complex, resulting in platelet dysfunction, macrothrombocytopenia and a severe bleeding phenotype, underscoring the essential role of this receptor in hemostasis [44–47]. (GP)Ib is considered a potential molecular target linking inflammation and thrombosis. For instance, blocking the receptor with a monovalent Fab-fragment in a murine stroke model of focal cerebral ischemia resulted in smaller infarct sizes, less ipsilesional occluded brain vessels, fewer activated microglia and macrophages and less TNF in the basal ganglia [48, 49].

GPVI, the main platelet receptor activator for collagen, is associated with platelet activation and endothelial inflammation [50]. Its signal is transduced *via* the immunoreceptor tyrosine-based activation motif (ITAM)-containing FcRγ-chain and a tyorisne phosphorylation cascade. Higher expression of (GP)VI was observed in acute coronary syndrome (ACS) compared with control groups. Reduced platelet counts in ACS patients are associated with an elevated surface expression of (GP)VI, suggesting a potential rise in activation and improved recruitment of platelets to the location of vascular injury [51]. Interestingly, mice lacking (GP)VI, either by genetic deficiency of the Gp6 gene or by antibody-mediated receptor depletion from the platelet surface, are profoundly protected from arterial thrombosis [52, 53] and experimental stroke [53], while having only a marginally impaired hemostasis. Notably, a Fab-fragment against human (GP)VI yielded promising results in a first clinical trial assessing safety and efficacy in acute ischemic stroke patients [54] and novel biologics are being developed [55]. Studies have found that soluble (GP)VI (s(GP)VI) was significantly decreased in AD patients compared to healthy controls, with s(GP)VI levels being positively correlated with cognitive status scores obtained by the Mini-Mental State Examination (MMSE) [56]. Additionally, blocking the (GP)VI receptor or its integrin significantly reduced amyloid aggregation in platelet cell cultures. While the loss of (GP)VI was shown to reduce the adhesion of amyloid-activated platelets in mouse arteries [57].

Human platelets express two additional adhesion receptors that use similar signaling machinery as (GP)VI, namely the C-type lectin-like type II transmembrane receptor CLEC-2 and FcγRIIA, the latter not expressed on murine platelets. CLEC-2 is expressed as a dimer on platelets, megakaryocytes, and at low levels on some myeloid cells and dendritic cells [58]. Podoplanin is the major known CLEC-2 ligand, which is also expressed outside the vasculature, including on lymphatic endothelial cells, the choroid plexus, and kidney podocytes, and is upregulated under inflammatory conditions on macrophages [59]. Lack of either CLEC-2 or podoplanin is associated with blood-lymphatic mixing, among other defects [59]. Increased tail bleeding times and reduced thrombus formation, including cerebral venous thrombosis [60], have been reported in most studies with CLEC-2-deficient mouse models, suggesting the presence of an additional ligand in the vasculature [61, 62]. The levels of CLEC-2 in human serum were significantly higher in AD patients compared to both control and Mild Cognitive Impairment (MCI) groups. Moreover, CLEC-2 levels were markedly higher in MCI patients compared to controls. Furthermore, a negative correlation between higher CLEC-2 levels and MMSE scores was shown [63].

GPIIb/IIIa (integrin αIIbβ3, CD41/CD61) is the most abundant integrin on the platelet surface and facilitates platelet adhesion and aggregation during hemostasis *via* its interactions with fibrinogen, but can also bind vWF as well as fibronectin and vitronectin [64–67]. Inhibiting the binding site of (GP)IIb/IIIa prevents the aggregation of platelets [68]. The functional deficiency of (GP)IIb/IIIa receptors in Glanzmann’s Thrombasthenia, a genetic bleeding disorder that causes platelet disfunction and diminished clotting [68], leads too several physiological effects including a lack of agonist-induced platelet aggregation in affected patients. People with this condition can display mucocutaneous bleeding, which may impact the (CNS) [69], this may suggest a potential link between platelet health and neurovascular homeostasis. Further research in this area may be promising. As previously mentioned, patients with AD who exhibited a faster rate of cognitive decline had higher baseline levels of (GP)IIb/IIIa compared to those with a slower rate of decline [15]. Besides (GP)IIb/IIIa platelets express four additional integrins, namely αvβ3 (vitronectin receptor), α2β1 (collagen receptor), α5β1 (fibronectin receptor), and α6β1 (laminin receptor) [70–72]. Virtually all platelet agonists trigger inside out signaling, which means the conformational change of the integrin from a low to a high affinity-state, thus, enhancing ligand binding, which, in turn, allows them to participate in outside in signaling (signaling of integrin receptor upon ligand binding) [70].

Most soluble agonists trigger platelet activation through G-protein coupled receptors (GPCRs). ADP and ATP are released from damaged endothelial cells and activated platelets and are essential amplifiers of platelet activation [73, 74]. Platelets express three purinergic receptors, P2Y1, P2Y12 and P2 × 1. P2Y1 and P2Y12 signal *via* the GPCRs (Gαq and Gαi, respectively), and are important for sustained platelet activation and thrombus formation. P2 × 1 is an ion channel causing Ca2 + influx upon activation, but with limited relevance for thrombus formation [75]. ADP triggers either a weak transient aggregation through intracellular calcium mobilization and protein kinase C activation or a potentially stronger response through inhibition of adenylyl cyclase activation and subsequent decreasing intracellular cAMP levels [76, 77]. Blocking of these receptors by binding thienopyridine is shown to prevent ADP-induced aggregation and thrombus formation as a result of platelet activation [77, 78]. Due to the central role of ADP signaling for platelet activation and as a consequence of the more restricted expression of P2Y12 (as compared to P2Y1) this receptor is a central target for anti-platelet strategies. Antagonizing P2Y12 with clopidogrel decreased platelet P-selectin, platelet-leukocyte aggregate formation [79] and reduced soluble CD40L and C-reactive protein in the context of cardiovascular disease [80]. P2Y antagonists were shown to suppress other platelet mediated pro-inflammatory reactions such as platelet leukocyte aggregate formation, leukocyte recruitment and activation, as well as generation of Reactive Oxygen Species (ROS) [81]. Likewise, P2Y12-null mice with carotid artery allografts for atherosclerotic treatment have altered plaque development [82]. Inhibition of P2Y12 during atherogenesis could also help to maintain cerebral blood flow and preventing the progression of neurodegenerative disorders as shown in rodent and non-human primate models [13, 83, 84]. Patients that have experienced a stroke face elevated post-stroke risk of developing cognitive disorders or dementia later in life [85, 86]. Ongoing studies are investigating the potential preventive benefits of antiplatelet medication targeting P2Y12 receptors [87]. Moreover, researchers found that P2Y12 was downregulated in microglia derived from brain regions with increased tau inclusion, despite an increase in microglia populations. This down-regulation was shown to occur before a significant accumulation of phosphorylated tau, even though the numbers of microglia were similarly elevated [88]. This points to alteration in P2Y12 receptor expression in AD, similar investigations into platelet P2Y12 receptor expression may be promising and warrant further investigation.

Aside from the GPRCs, platelets possess damage-associated molecular pattern receptors, such as Toll-like receptors (TLR), and immunoreceptor tyrosine-based inhibition motif (ITIM)-containing immunoglobulin receptors and C-type lectin receptors (CLRs) [89]. The main TLRs in platelets are TLR-4 and TLR-2, which participate in inflammatory signaling by triggering cytokine production upon systemic inflammation. For instance, TLR4loxP/Pf4-cre mice lacking TLR4 in platelets and megakaryocytes demonstrated decreased IL-6 concentration in the serum and reduced injury severity following hemorrhagic shock and resuscitation [90]. Likewise, activation of platelet TLR2 was observed to increase inflammatory responses of human umbilical vein endothelial cells (HUVECs) and promoted their adhesion under static and flow conditions [91]. Both TLR-4 and TLR-2 agonists trigger platelet activation through NF-κB [92] to induce the release of pro-inflammatory cytokines like IL-1, IL-6, and IL-8 [93]. Moreover, TRL-4 and TLR-2 expression levels have been associated with severe disease states in inflammatory responses such as arterial thrombosis, acute coronary syndrome, and pulmonary hypertension [94–99]. Elevated TLR-4 and TLR-2 mRNA and protein levels were observed in peripheral blood mononuclear cells (PBMCs) for late-onset Alzheimer’s Disease (LOAD) patients [100]. Additionally, it was found that platelet expression of TLR-4 was upregulated in AD subjects compared to control [73, 101]. Further research into platelet TLR-4 and TLR-2 could prove promising, especially when considering the effect of Aβ) on inducing platelet TLR expression [73].

Platelets express several ITIM-containing receptors, namely PECAM-1 (also referred to as CD31), G6b-B, triggering receptors expressed on myeloid cells-like (TREM-like) transcript-1 (TLT-1), and carcinoembryonic antigen-related cell adhesion molecule 1 (CEACAM1) and CEACAM2 [102]. The canonical mode of action of ITIM-containing receptors is positioning phosphatases in close proximity to ITAM-containing receptors, such as (GP)VI or CLEC-2, to dampen ITAM-signaling. Consequently, platelets lacking PECAM-1 or G6b-B display potentiated platelet activation in response to GPVI stimulation [103, 104]. Of note, either knockout mouse line displays prolonged tail bleeding times, which is, however, attributed to the loss of endothelial PECAM-1 [103] and reduced platelet counts in the absence of G6b-B [105], respectively. Studies have noted that no significant changes were identified in platelet PECAM-1 levels between patients with MCI and AD [106, 107]. However, PECAM-1 was shown to be elevated in the serum [108] and endothelial microparticles in the peripheral plasma of AD patients [109].

Initially identified as the inhibitory analog of the triggering receptor expressed on myeloid cells-1 (TREM-1) family of immune receptors, (TREM)-like transcript-1 (TLT-1) was hypothesized to negatively regulate TREM-1 activity, yet its expression in blood cells is restricted to megakaryocytes and platelets [110], which have no detectable TREM-1. TLT-1 is localized specifically in α-granules in resting platelets, and is upregulated on the surface after activation [110]. Platelet activation with thrombin results in the shedding of the ectodomain of TLT-1, referred to as soluble TLT-1 (sTLT-1), which is a competitive inhibitor of TREM-1 and has anti-inflammatory properties [111]. Indeed, sTLT-1 levels were substantially elevated in plasma from septic patients compared with healthy individuals [112].

CEACAM1 and CEACAM2 get upregulated on the surface of activated platelets and both receptors attenuate ITAM-mediated responses while positively regulating integrin mediated responses [102]. In vivo, their main function appears to be limiting thrombus growth [102].

Platelets express receptors that contain an immunoreceptor tyrosine-based switch motif (ITSM) in their cytoplasmic domain and belong to the signaling lymphocytic activation molecule (SLAM) family [113]. Under steady-state conditions murine megakaryocytes and platelets express CD150 and CD84 [114, 115], while inflammatory conditions result in the expression of CD48 in a subset of murine megakaryocytes [116]. Elevated levels of CD48 gene expression has been observed in several AD disease groups, with the over expression of CD48 and CD40 being connected to tau aggregation [117]. Human platelets express SLAMF6 (CD352) and SLAMF4 (CD244/2B4) in addition to CD150 and CD84 [118]. SLAM becomes tyrosine phosphorylated in an aggregation dependent manner and SLAM-deficient platelets exhibit subtle defects platelet aggregation in vitro and a slightly delayed arterial thrombus formation in vivo [114]. Upon platelet activation, CD84 is shed from the platelet surface [119], however, CD84 is dispensable for classical platelet signaling pathways and hemostasis or thrombosis [115]. Remarkably, however, platelet-derived soluble CD84 acts on CD4 + T cell CD84 leading to enhanced CD4 + T cell motility in vitro and aggravated infarct growth following experimental stroke [120].

The A_2A_ adenosine receptor plays an important role in platelet aggregation. Adenosine acting on adenosine receptors on platelet cell surfaces plays a role in inhibition of aggregation *via* intracellular camp signaling. Knock out of the A2A gene (A2bAR) has been linked to increased ADP receptor mediated activation and aggregation in platelets [121]. The A_2A_ adenosine receptor, present on peripheral platelet and through cortical brain matter, is significantly upregulated in AD patients [18].

CD36, another platelet surface receptor at the crossroad of inflammation, serves as a signaling hub protein that links to oxidized low-density lipoprotein (LDL), long-chain fatty acids, thrombospondin-1, and fibrillar Aβ [122, 123]. CD36 interaction with oxLDL was shown to sensitize platelets to low doses of agonists [124] and participate in oxidative stress, hyperlipidemia, inflammation, and pathological thrombosis [125]. Additionally, CD36/oxLDL interaction is shown to induce secretion of vimentin and the release of pro-inflammatory cytokines (such as TNF and IL-6) in activated macrophages, which places CD36 as an active player in the onset and progression of atherosclerosis, ranging from endothelial dysfunction to thrombus formation [125].

Similarly, Piezo1 is a mechanosensory membrane-bound ion channel receptor that participates in inflammatory processes [126]. In particular, depletion of endothelial Piezo1 was shown to decrease inflammatory signals and activation of integrin with regression of atherosclerosis [127]. Piezo1 plays a crucial role in regulating the physiological response of platelets by controlling ion flux across the membrane. Inhibiting Piezo1 has been shown to decrease the severity of arterial thrombosis and stroke in mice [128]. Additionally, platelets can sense surface stiffness during the process of clot formation, through Piezo1, which directly influences platelet adhesion and spreading *via* Rac1 and actomyosin activity [129, 130]. Increased stiffness of the underlying clotting substrate is then correlated to increased platelet activation, again potentially linking platelet activation to conditions like atherosclerosis, a risk factor for dementia [129]. Piezo1 expression on microglia have been shown to up regulate upon exposure to Aβ plaque-associated tissues due to its increased rigidity [131]. Further experiments are needed to shed light on the specific contributions of Piezo1 to platelet activation, aggregation, and response to inflammation in the context of dementia.

Thromboxane (TxA2) is an important second wave mediator that is produced and released by stimulated platelets [132, 133]. The conversion of arachidonic acid into TxA2 is mediated by cyclooxygenase-1 and can be irreversibly inhibited by acetyl salicylic acid (ASA), still the gold standard drug for prevention of cardiovascular syndromes [134]. Secreted thromboxane activates further platelets *via* the Gq and G12/13-coupled thromboxane-prostanoid receptors TPα and TPβ thereby promoting thrombus formation [133]. Ablation of TxA2 receptors delays platelet aggregation in response to collagen, prolongs bleeding time and results in thrombus instability [133].

Thrombin, also known as factor IIa, is a serine protease that is the most potent soluble platelet agonist and is also responsible for converting plasma fibrinogen into fibrin to stabilize the platelet-rich aggregate [135]. Thrombin activates platelets through protease-activated receptors (PARs) that convert an extracellular proteolytic cleavage event, which unmasks a new N-terminus acting as tethered ligand, into an intracellular signal *via* Gq, G12/13 [136]. Mouse and human platelets differ in their PAR expression, as human platelets express PAR1 and PAR4 [137], while mouse platelets express PAR4 and PAR3, the latter not transducing signals by itself, but rather functioning as a cofactor for the cleavage and activation of Par4 by thrombin [138]. PAR1 mediates human platelet activation at low thrombin concentrations, whereas PAR4 contributes to sustain platelet activation at high thrombin concentrations [139]. Of note, PARs are also expressed in a variety of other cells ranging from endothelial cells, smooth muscle cells and fibroblasts to leukocytes [140]. Elevated thrombin concentration in the cerebrospinal fluid (CSF) of intracerebral hemorrhage (ICH) patients is associated with poor functional outcomes [141]. In mouse models with induced intraventricular hemorrhage, thrombin played a major role in inducing hydrocephalus, ventricular wall damage, and periventricular Blood Brain Barrier (BBB) disruption. Inhibition of PAR1 reduces cerebral edema and ventricular dilation [142]. Thrombin’s involvement in blood-CSF barrier disruption appears to be predominantly mediated *via* the downregulating of VE-cadherin, increasing vessel permeability and contributing greatly to the development of hydrocephalus in rats. Blocking the PAR1 pathway was shown to increase VE-cadherin levels and reduced thrombin-induced hydrocephalus [143, 144]. Thrombin-induced hydrocephalus involves the recruitment of macrophages, microglia, and neutrophils, emphasizing the intricate role of thrombin in CNS injury following injury or severe bleeding episodes [145, 146].

### Platelet granules and microvesicles

**Fig. 2:**
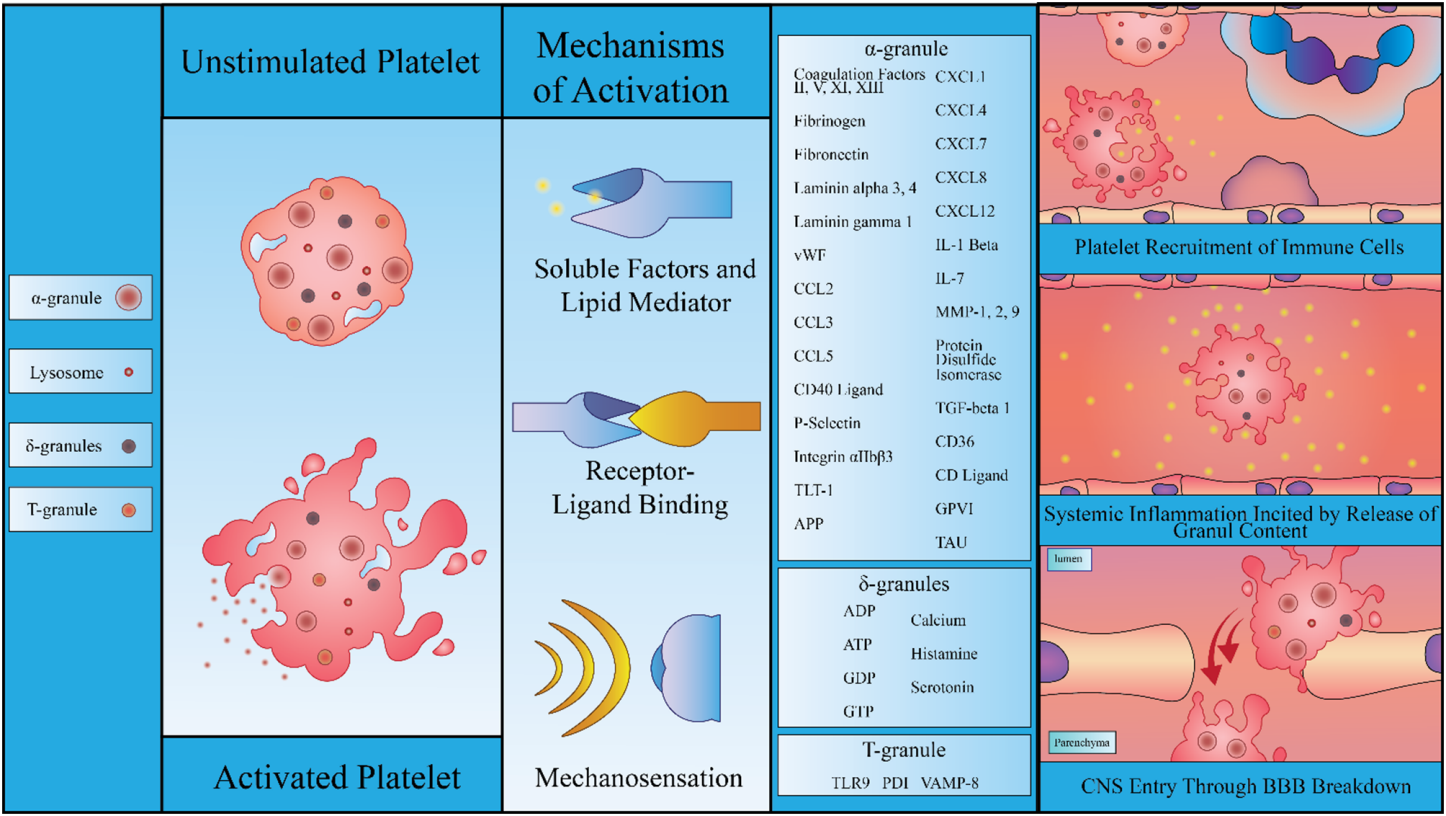
Dynamic Impact of Platelet Activation and Subsequent Granular Content Release. The figure depicts the anatomy and contents of both inactivated and activated platelets, highlighting the common mechanisms of platelet activation, including chemokine/cytokine detection, receptor ligand binding, and mechanosensation. Following activation platelets release, a wide variety of soluble factors from various internal granules that influence their surrounding environment. These factors have the potential to induce broad systemic and local effects on inflammation. They can promote immune cell recruitment to inflammatory sites, induce systemic inflammation by secreting platelet cytokines and chemokines, and facilitate platelet infiltration into the parenchyma surrounding blood vessels through breaks in the BBB. This process may activate additional platelets and exacerbate dysregulation in disease states such as AD

The strong regulatory potential of platelets in hemostatic and inflammatory events also stems from their ability to secrete granular constituents [147]. Platelets harbor 3 major granules: α-granules, dense granules, and lysosomal granules, in addition to peroxisomes and lysosomes [147]. Recently a fourth type of granule, T granules, was described for its role in platelet secretion of protein disulfide isomerase (PDI), which play a role in platelet aggregation and thrombus formation [148, 149]. Figure 2 illustrates the platelet granules, their contents, and the extensive effects they exert in systemic inflammation.

α-Granules are the most abundant granule type in platelets, with 50–80 per cell, and their development begins in the megakaryocyte [147, 150]. During platelet biogenesis, these intracellular compartments are passed down by the megakaryocyte to the platelet where their development continues [151]. α-Granules harbor a diverse array of both soluble and surface bound proteins which are released upon activation [147, 151]. Receptors include several integrins and immunoglobulins amongst others commonly found on the cell surface of resting platelets [151]. Platelet α-granules secrete a variety of coagulation mediators including fibrinogen, vWF and coagulation factors V, XI, and XIII [35, 37, 151]. They also harbor inflammatory mediators such as PF4 (CXCL4), CD62P, CD40L, RANTS (CCL5), transforming growth factor (TGF)-β, and macrophage inflammatory protein (MIP)-1α along with a diverse set of chemokines [35, 37, 151]. Among those factors is P-selectin, which is translocated to the cell surface upon platelet activation, where it mediates rolling and interaction with neutrophils and monocytes [152]. Upon vascular inflammation, endotoxin-induced platelet interactions were reported to be dependent on the increased expression of endothelial P-selectin interacting with platelet PSGL-1 [153]. In the context of atherosclerosis, a platelet-specific knockout of P-selectin reduces lesion development by 30% in apoE−/− mice [154]. The same study infers that platelet granules, which carry P-selectin, facilitate leukocyte-leukocyte interactions in a flow chamber study [155] and, thus, have the potential to enhance the infiltration of monocytes into atherosclerotic lesions [154]. Similarly, RANTES or CCL5 are released by α-granules and are binding to endothelial cells to enhance atherogenic T lymphocyte recruitment [156, 157].The α-granular contents release during platelet activation at sites of vessel wall injury and thus play an important role in hemostasis, inflammation, wound repair, and the pathogenesis of atherosclerosis and similar vascular conditions [158].

Interestingly, it has long been known that APP is found in megakaryocytes and subsequently in the α-granules of platelets in relatively high concentrations (1.1 ± 0.3 µg/10^8^ platelets) and is released upon platelet degranulation [159]. Additionally, elevated levels of high molecular weight tau protein, which correspond to the oligomeric form of the protein, in platelets have been identified in AD patients [160]. Additionally, both full-length and truncated alpha-synuclein, critical for calcium-dependent granule release and a hallmark for Parkinson’s Disease (PD) are present in platelets. However, no correlation was found between platelet concentration and disease presence or severity in PD [161, 162].

Dense granules are less abundant, with only 3–8 in a given platelet [147, 150], and contain serotonin, histamines, and nucleotides such as ADP, ATP, GDP, and GTP, amongst other factors known to mediate arterial inflammation [35, 37]. Particularly, histamine was shown to modulate neutrophil-platelet interactions in response to coronary micro-thrombosis, as histamine deficiency proved to promote micro-thrombosis, and deteriorate cardiac function post-acute myocardial infarction, by enhancing platelets/neutrophil function [163]. Platelet serotonin was found to promote the recruitment of neutrophils in acute inflammation, supporting an important role for platelet serotonin in innate immunity [164]. A study showed that upon inflammatory stimulation, neutrophil extravasation into the lung, peritoneum, and skin wounds were reduced in mice lacking non-neuronal serotonin [164]. ADP is a platelet agonist released upon activation. ADP is able to induce platelet aggregation through binding members of the P2Y receptor family, such as P2Y12 discussed above.

Lysosomal granules are one of the least abundant granule types [147]. They assist platelets in conducting phagocytic and cytosolic processes through hydrolases such as glycohydrolases and proteases like carboxylpeptidase and cathespin types D and E which also play a role in platelet-derived inflammatory reactions [165].

Platelet-derived extracellular vesicles (PEVs) are the most abundant microvesicle (MV) in the circulation, making up between 60 and 90% of the MV population at any given time. PEVs production can occur spontaneously or be activated by exposing platelets to shear stress, glycoprotein IIb/IIa, or exposing platelets to soluble agonists such as collagen [166, 167]. Increased numbers of PEVs are shown to induce increased levels of ROS and reduce endothelial nitric oxide synthase (eNOS) and superoxide dismutase (SOD) activity. This results in decreased nitric oxide levels and reduced thickness of the endothelial outer layer [167]. PEVs are shown to be elevated in AD patients [168] and increased number of PEVs containing Aβ1–42, were linked to an earlier onset of dementia and was correlated with cognitive decline in PD [169], both demonstrating the importance of PEVs in neurodegenerative conditions. An experiment conducted by Tateno et al. shows that PEV glycans were profiled for both AD and control patients. Both groups showed a distinct glycan profile, with AD patients showing increased signaling of Mannose-binding lectin and a significantly increased number of platelet-derived exosomes, another platelet-derived extracellular vehicle [168, 170].

### Lipid mediators

Lipid mediators secreted by platelets, play a vital role in inflammatory events due to their ability to regulate immune responses and modulate the inflammatory process [171] through the induction of vasodilation, leukocyte recruitment, and platelet activation [172]. For instance, thromboxane A2 (TxA2), derived from arachidonic acid and primarily synthesized within platelets, acts as a potent vasoconstrictor and platelet activator [134]. TxA2 has been implicated in promoting inflammation and exacerbating rheumatoid arthritis, inflammatory bowel disease, and asthma amongst other conditions [134]. Elevated levels of TxA2 have been observed in the synovial fluid of rheumatoid arthritis patients and correlated with disease activity [173]. Additionally, TxA2 levels were significantly increased in COVID19 patients, suggesting that interactions between immune-inflammatory pathways and platelet hyperactivity participate in the pathophysiology [174]. COVID19 has been shown to exacerbate AD symptoms potentially *via* modulating the expression of inflammatory proteins and compounding the already dysfunctional neurons by causing a proinflammatory microenvironment in areas with hallmark tau and Aβ accumulation [175, 176]. This is further exacerbated by changes in gene expression in platelets derived from patients who contract the virus, potentially leading to a hyperactive state that contributes to increased thrombus formation compounded by increased platelet aggregation *via* platelet-platelet and platelet-leukocyte interactions [177].

Platelets also secrete 20-Hydroxyeicosatetraenoic acid (20-HETE), which promotes the secretion of insulin from pancreatic β-cells as well as influences hypertension and vasoconstriction, indicating that platelets may play a role in the regulation of metabolism [178, 179]. Population studies have linked mutation in the enzymes that generate 20-HETE to AD [179]. Due to the involvement of platelet secreted 20-HETE in vascular regulation and its link to AD in other cell types, furth examination into potential alterations of platelet derived 20-HETE secretion in neurodegenerative conditions may prove promising. Platelet Activating Factor (PAF), another lipid mediator, promotes leukocyte adhesion and activation, and mediates the release of other inflammatory mediators [180]. The link between PAF and systemic inflammatory response syndrome (SIRS) in the context of acute pancreatitis has been previously established. In acute pancreatitis, the release of proinflammatory cytokines, anti-inflammatory cytokines, and tumor necrosis factor α contributes to the development of SIRS and systemic multi-organ failure [181]. PAF binding to platelets was shown to be significantly reduced in patients with AD and multi-infarct dementia (MID) when compared to both the old control group and younger subjects in the study. No significant difference was observed between AD and MID groups [182]. Platelet-derived lipid mediators such as TxA2 and PAF contribute to the complex orchestration of inflammatory events and help regulate the immune response in various inflammatory conditions.

### Chemokines and cytokines mediators of inflammation

Activated platelets release various chemokines including a diverse variety of C-X-C motif ligand such as CX3CL1, CXCL4, CXCL7, CXCL8, CXCl12, and CXCL16 as wells as C-C motif ligand including CCL2, CCL3 and CCL5. The release of these cytokines plays a major role in regulating the activity of the innate immune system and plays a part in inflammation through the upregulation of ROS production [36, 183].

CXCL4 (Platelet factor 4 or PF4) is the most abundant chemokine. It has diverse functions in cell differentiation, immune cell activation in monocytes, neutrophils, and lymphocytes, phagocytosis induction, ROS generation, and hemostasis [184, 185]. CXCL4 deposition in capillary endothelial tissues has been linked to the severity of lesions in atherosclerosis [186, 187]. The cell receptor CCR5 forms a heterodimer with CXCL4, and this dimmer plays a role in CD4 T-cell homing to areas of atherosclerotic damage [183] in addition to leukocyte recruitment [188]. It also increases cytotoxicity and cytokine production in CD8 T-cells [189]. CXCL12 (Stromal cell-derived factor 1, SDF-1a) and its major receptor CXCR4 regulate megakaryopoiesis, platelet function, and paracrine functions, such as cell proliferation and differentiation. Notably, CXCL12 alters platelet-mediated recruitment of progenitor cells to sites of injury and is shown to be elevated in patients experiencing acute myocardial infraction with a positive correlation to the number of circulating progenitor cells [190]. Exposure to CXCL16 induces platelet activation and subsequent adhesion to sites of injury following binding to a CXC motif receptor [191]. Platelet CCL5 (RANTES) secretion is stimulated by monocyte tethering to endothelial cells via P-selectin (CD62P). When CCL5 is released from the α-granules, it binds to receptors such as CCR5, CCR3, and CCR1, causing monocytes and T lymphocytes to stop and transmigrate into inflamed endothelium [192]. RANTES expression is also shown to increase when platelet interact with B-cells and IgG synthesis [193]. CCL3 secreted by platelets, also known as macrophage inflammatory protein, is involved in the recruitment of polymorphonuclear leukocytes [194–196]. In one study, it was found that CCL3 influenced the invasive capability of certain cancer cell types through its interaction with CCR5 on endothelial cells [194]. Additionally, our laboratory has shown that leukocytes adhere to vessels and this adhesion contributes to cerebral blood flow reduction and reduced cognitive ability [13, 84].

Platelets release a variety of matrix metalloproteinases (MMPs), which are a family of zinc-containing endopeptidase, which have a wide array of effects on tissue healing and remodeling [197, 198]. MMP-1, also known as human fibroblast collagenase, participates in a diverse set of roles including collagen fibril turnover, cleavage of cell surface substrates and non-matrix substrates, and potentially development and wound repair [198]. Additionally, MMP-1 was shown to activate Protease-Activated Receptor-1 (PAR1) on nearby platelets. Blockage of the MMP1-PAR1 pathway was shown to reduce thrombogenesis and thrombosis in guinea pig models [199]. MMP-2 and MMP-9, also known as gelatinases, similarly have roles in collagen degradation and coagulation regulation [200]. MMP-2 was shown to activate platelets in a similar manner to fibrinogen by interacting with the αIIβb3 integrin as well as cleaving PAR1 in a similar fashion to MMP-1 [201]. Inhibition of MMP-2 was also shown to reduce thrombus formation under flow. Unlike MMP-1 and MMP-2, inhibition of MMP-9 is believed to stimulate platelet aggregation [202]. MMP-14, unlike the previously mentioned MMPs was shown to have a role in resolving thrombi and thrombi developed under MMP-14 inhibition were shown to be larger [202]. Hence, by modulating platelet aggregation/thrombus formation platelet-derived MMPs could affect cerebral blood flow and thereby have an impact on neurodegenerative diseases.

## Crosstalk at the crossroads of inflammation: platelet crosstalk under inflammatory conditions

Platelet aggregation is a dynamic process where platelets in flowing blood continually link, shift, deform, and detach from the inner surface of a developing thrombus, while only a small proportion creates stationary adhesion contacts [203]. Platelet adhesion under high shear conditions require a deceleration *via* (GP)Ibα-vWF (immobilized on endothelial cells, exposed collagen or bound to platelets) [204]. Ultimately an irreversible phase of aggregation dependent on integrin αIIbβ3 [204], which requires platelet activation and cross-links platelets *via* vWF and/or fibrinogen [203]. Platelet activation not only results in functional up-regulation of integrins, but also triggers the release of granule content. In the context of the formation of three-dimensional aggregates, second wave mediators are crucial to facilitate platelet activation in adjacent layers, among them ADP plays a key role [205]. Interestingly, hyperreactivity to low-dose ADP in human patients, who were free of cardiovascular disease and any anti-platelet therapy, is associated with future arterial thrombosis [205].

### Platelet-endothelial crosstalk

Platelet rolling plays a crucial role in both inflammatory and hemostatic processes and involves force-regulated and rapidly reversible transient interactions between receptors on the endothelial and platelet cells surfaces [72, 206]. Platelets are able to travel across the surface of blood vessels through a series of low affinity bonds with thrombogenic extracellular matrix (ECM) protein collagen via their (GP)VI receptors [72]. Additionally, P-selectin was shown to play a role in mediating platelet rolling behavior in addition to facilitating platelet rolling as aggregates with leukocytes on the endothelial surface of capillaries following activation [207]. Additionally, platelet adhesion to the endothelial wall is shown to increase substantially when shear forces are applied, P-selectin is rapidly expressed on both platelet and endothelial cell surfaces upon stimulation [207]. Platelets interact with exposed ECM components such as collagen, fibrinogen, and vWf lead to irreversible interactions [208]. Interactions with these components help to initiate the coagulation cascade, which in turn generates important platelet agonists such as thrombin which not only mediates platelet aggregation, but also activation of several cell types, including monocytes, dendritic cells, and T cells, *via* a family of thrombin protease-activated receptors (PARs) [209]. Endothelial cells express the ectonucleotidase CD39 on their surface which can hydrolyze proinflammatory and prothrombotic molecules such as ADP and ATP, released from activated platelets. CD73 plays a similar role in hydrolyzing AMP, the products of these reactions serve to further activate platelets [210]. In atherosclerotic mice models, platelet adhesion to endothelial cell walls preceded the development of atherosclerotic lesion, binding preferentially to lesion prone cites, regardless of age, further highlighting the role of these platelet interaction in cardiovascular disease development [211].

The local shear rate of blood plays a significant role in determining platelet adhesion to the subendothelium [212]. At low shear rates, which occur during venous flow (100–1000 s^− 1^), platelets can interact with a variety of ECM components including collagen *via* their (GP)VI and α2β1 receptors, fibronectin *via* their integrin αIIbβ3, αVβ3, and α5β1 receptors, and laminin *via* its α6β1 receptor [212]. At high shear rates (1000–4000 s^− 1^), initial adhesion relies on platelet (GP)Ibα and vWF interaction [212]. vWF can take two distinct conformations when exposed to different shear forces. Under low shear forces, vWf has a globular shape, which is believed to limit the number of binding sites exposed to the environment [213]. Under high shear forces, those exceeding (3,000 s^− 1^) for surface bound vWF and (5,000 s^− 1^) for circulating vWF, the molecule becomes “stretched” as interactions between its monomeric units are pulled apart by the drag generated by rushing blood. This allows for an increased number of available binding sites [214–216].

### Platelet-leukocyte crosstalk

Platelet-leukocyte crosstalk highlights the versatility and interconnectivity of platelet-cell interactions serving as a regulatory and bridging force for both the adaptive and innate immune systems. Inflammatory conditions promote the aggregation of platelets near the vessel wall and raise the chances for interactions with leukocytes, such as neutrophils and monocytes. In turn, this increases the likelihood of temporary platelet-leukocyte interactions, which can become permanent during vascular inflammation when these cell-types become activated [217]. Platelet–leukocyte interactions are initiated when leukocyte PSGL-1 binds to P-selectin, expressed on the surface of activated platelets [218]. Platelet–leukocyte interactions mediated *via* leukocyte tethering to platelet P-selectin triggers both a rapid response *via* rapid β2 integrin activation and a delayed response which is mediated by alterations in gene expression necessary for leukocytes to take on an inflammatory phenotype [219]. Examples of this slow response include P-selectin-related NF-κß translocations, such as the ones involved in monocyte chemotactic protein-1 (MCP-1) and Interleukin (IL-8), and COX-2 derived eicosanoids production in monocytes [220, 221].

During infection, inflammation, and thrombosis, platelets and neutrophils interact with each other, thereby modulating one another’s functions [222]. Platelets initially protect the vessel wall from neutrophil-mediated sequelae [223, 224]. However, subsequent interaction of Mac-1 integrin on recruited neutrophils with (GP)Ibα on platelets triggers thrombosis that is responsible for vessel occlusion and organ damage, as shown in a mouse model for thrombotic microangiopathy [224]. Moreover, platelet integrin αIIbβ3 also plays a vital role in regulating platelet-leukocyte interactions. Not only does integrin αIIbβ3 serve as a binding partner to integrin αLβ2 (Mac-1) on neutrophils *via* a bridge of soluble fibrinogen [225], but it also has been shown to trigger outside-in signaling upon cytokine-binding such as CCL5, CXCL12, and CX3CL1 [226].

Increased platelet-neutrophil interactions have been identified in an ever-increasing variety of immunothrombotic and thrombo-inflammatory disease settings [28, 227–229]. Platelets recruit neutrophils by the release of cytokines and by exposing the neutrophil PSGL-1 ligand P-selectin and by exposing phosphatidylserine (PS) [229]. In line with this, mice lacking cyclophilin D in platelets had less neutrophil infiltration and smaller infarct volumes following experimental focal ischemia, which was attributed to the abolished PS exposure in CypD-/- platelets [230]. Platelets not only recruit neutrophils, they also promote the formation of neutrophil extracellular traps (NETs), which are extracellular webs made of DNA fibers embedded with neutrophil granule proteins that can exert immunomodulatory effects by activation and differentiation of macrophages, dendritic cells and T cells [231, 232]. The high-mobility group box 1 (HMGB1), which is stored in platelet α-granules and elevated in stroke patients, contributes to cerebral damage by promoting NET formation [233]. Likewise, inhibition of NETs by neonatal NET-inhibitory factor (nNIF) [233] or DNAse [234] reduces infarct volumes after experimental stroke. NETs, in turn, are also a prominent driver of thrombosis [28, 227–229], induce platelet-granule exocytosis, reactive oxygen species (ROS) production by the NADPH oxidase NOX2, and secretion of the proinflammatory chemokine IL-8 and the B-cell-activating cytokine BAFF [235]. Of note, many known NET inducers, such as IL-1β, IL-8, TNF, and G-CSF are increased in COVID-19 patients [236]. Additionally, histones, which are highly alkaline proteins involved in the storage and organization of genetic material in the cell nuclei, are also major components of NETs and have been shown to be highly thrombotic proteins on their own [237, 238]. The formation of platelet-neutrophil aggregates induces an integrin-mediated outside-in signaling into neutrophils, which in turn stimulates chemokines for ROS production. At basal levels, ROS function as signaling molecules that regulate cell growth, the adhesion of cells toward other cells, differentiation, senescence, and apoptosis. However, elevated ROS levels can be injurious, because of their ability to oxidize protein and lipid cellular constituents, as well as damage the DNA. Under inflammatory conditions, platelets are exposed to phagocyte-dependent “burst-like” production of high quantities of ROS that are known to modulate platelet function [239, 240]. For instance, stored platelets with lower levels of intra-platelet ROS exhibited decreased levels of important platelet pro-inflammatory molecules such as P-selectin and CD40L [241].

Monocytes have traditionally been regarded as dominant cell types during the resolution of inflammation and platelets are known to induce various responses in monocytes in regard to pro- and anti-inflammatory phenotypes [242]. For instance, platelet P-selectin and monocyte PSGL1 binding results in the upregulation of monocyte proinflammatory surface markers (e.g., CD40), migration (CD11b/CD18), and procoagulant tissue factor (TF), the initiator of the extrinsic arm of coagulation. In addition, monocytes exposed to platelets secrete proinflammatory cytokines (TNF, MCP-1, IL-1β) and exhibit a proinflammatory transcriptome [242]. How activated platelets induce a proinflammatory monocyte phenotype, affecting the inflammatory response, has been described in rheumatoid arthritis [243], cardiovascular disease [243], and COVID-19 [244]. However, a different study indicated that human platelet-conditioned media, through prostaglandin E2 receptor 4 (EP4) activation by prostaglandin E2 (PGE2), is responsible for anti-inflammatory phenotypic changes in macrophages in vitro that are associated with in vivo atherosclerosis regression [245] which has led to the conclusion that platelets can elicit contrasting responses depending on the underlying pathology, site of inflammation, and experimental model employed [246].

Platelets also play a role in the adaptive immune system responses by participating in cross-talking with T and B cells. RANTES secreted by platelets is shown to recruit T cells to sites of inflammation [247]. Activated platelets interact with both helper and cytotoxic T cells, the results of which are often cyclic activation and recruitment of the two cell types. T cells are recruited by the platelet’s chemokine secretion and the T cells in turn activate platelets the CD40-CD154 interaction causing additional cytokine secretion. Platelets, much like dendritic cells, can activate peripheral B cells and subsequently induce production [248]. Platelets can induce B cell isotype switching *via* the CD154 receptor, which is expressed on platelets [249].

## The platelet problem: the role of platelet in Alzheimer’s Disease and related dementias

**Fig. 3:**
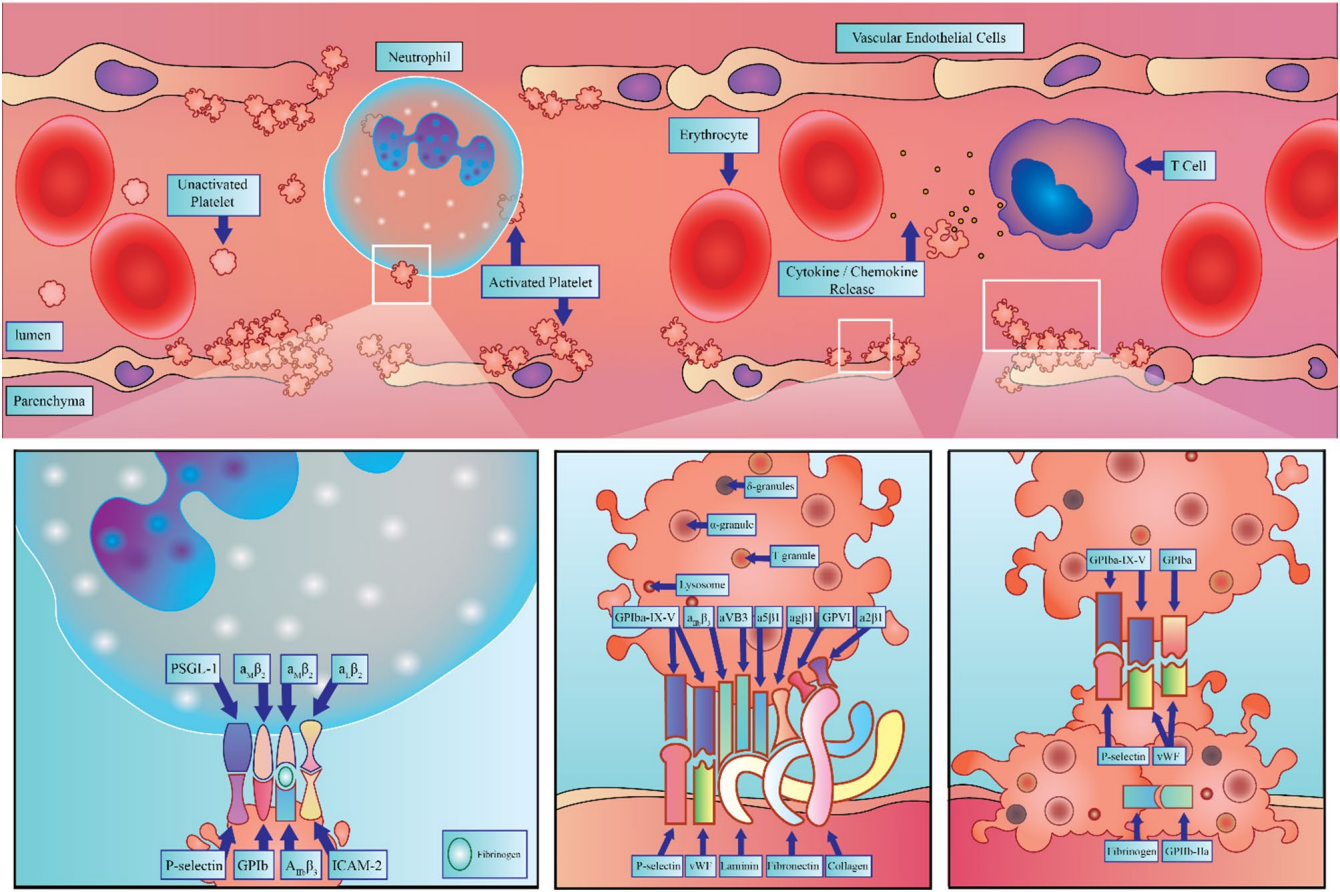
Overview of Platelet Activity in blood vessel from neurodegenerative conditions. The figure depicts platelets in the vascular environment, highlighting the diversity of interactions they can participate in including platelet-neutrophil, platelet-endothelial, and platelet-platelet crosstalk. Additionally, breakdown of the blood-brain barrier and potential platelet infiltration is shown. The inlets highlight the most prominent receptor ligand bindings that occur in platelet-neutrophil, platelet-endothelial, and platelet-platelet crosstalk, from left to right, in inflammation

Platelets are intimately involved in the dynamic interactions that take place during low level chronic inflammation [183]. Figure 3 below presents a summary of various platelet-mediated activities discussed in this paper within the context of neuroinflammation. Platelet membrane-bound and -derived factors contribute to the activation of immune cells, endothelial dysfunction, and tissue remodeling, thus promoting inflammatory conditions [250]. Given the adverse impact of inflammatory conditions on severe neurological disorders, platelets may play a significant role in the mechanisms that underlie several forms of dementia including Alzheimer’s Disease (AD), Frontotemporal Dementia (FTD), Vascular Dementia (VaD) and Lewy Body Dementia (LBD).

Significant insights have been derived regarding the role of platelets in age-related neuroinflammation and cognitive impairment. Patients with increased levels of platelet activation biomarkers, such as activation detected by the active conformation of glycoprotein (IIb-IIIa αIIbβ3) complex and P-selectin exposure, experienced a faster rate of cognitive decline, regardless of age, gender, education level, baseline MMSE score, and cardiovascular risk factors [15]. This suggests that platelet activation could be used as a potential predictive biomarker for cognitive decline in such patients [15]. Additionally, age is a significant factor in both platelet and neurological health. In general, platelet aggregation increases around midlife, with significantly higher rates of platelet reactivity in patients in their early 40s than in their 20s. This change is driven by alterations in the transcriptome of platelets and characterized by increased platelet reactivity to ADP and collagen, increased intracellular signaling potentially like that of the mammalian target of rapamycin (mTOR), and increased stability in vWF binding [251–256]. Recent studies have shown that older mice treated with blood plasma containing platelets from young mice in addition to increased levels of platelet factor 4 (PF4) exhibited reduced neuroinflammation in the hippocampus at both the transcriptional and cellular level, resulting in improved cognitive function [257–259]. Additionally, treatment with the longevity factor klotho, which is known to decline in aging, has been shown to increase cognition in aged mice [260]. Klotho has also been shown to increase multiple platelet factors, mainly PF4, in the plasma, which has also been linked to enhanced cognition [258].

One prominent hypothesis suggests that the complete blood counts influences brain function and cognitive performance since an elevated platelet count inhibited the effect of functional changes in the left precuneus on executive function in 22 patients with MCI [261]. On the contrary, a separate study analyzing hematological and inflammatory parameters from 97 patients undergoing dementia evaluation found no statistically significant variances in white blood cell count, platelet count, neutrophil-to-lymphocyte ratios, platelet-to-lymphocyte ratio, or CRP levels between the control and dementia groups including FTD, VaD, and AD [262]. Another study involving 410 patients broken into VaD, AD, and control patients revealed decreased mean platelet volume and platelet distribution width in both VaD and AD groups [263] suggesting that platelet numbers were reduced in neurodegenerative disease conditions. Further investigation into this matter may be necessary.

Platelets have been implicated in the aggregation of Aβ peptides, a hallmark of AD. Platelets readily express APP, with early studies attributing as much as 90% of circulating Aβ peptides to the release of platelet al.pha granules, which come pre-packaged from the megakaryocyte [264–266]. In mice, platelet-derived Aβ sever a notable source for Aβ in the brain tissue following several micro-thrombin events due to platelet accumulation and activation at sites of injury [265, 267]. It is recognized that platelets undergo activation due to Aβ peptides in peripheral circulation, which are themselves released from activated platelets [268]. This cycle could induce repeated platelet activation, prompting additional Aβ release and subsequently fueling chronic inflammation by raising levels of circulating cytokines and chemokines [195]. This process can influence the aggregation and accumulation of Aβ plaques throughout the body and is thought to contribute to the neuroinflammatory response observed in many dementia subtypes [269–271]. Circulating Aβ40 was shown to result in elevated production of ROS and superoxide production, key characteristics of mitochondrial dysfunction. Enhanced mitochondria disfunction, in turn, triggers platelet-mediated Aβ40 aggregate formation through (GP)VI-mediated ROS production and resulted in enhanced integrin activity [272]. It has also been shown that circulating Aβ is able to cross the BBB [273, 274], this data suggests that platelets themselves may trigger neuroinflammation [273]. Additionally, there is a link between reduced levels of serum Aβ-albumin complexes, which aid in the removal of Aβ from the brain, decreased levels of CSF Aβ42, increased levels of CSF phosphorylated-tau, and a higher incidence of AD [275]. Furthermore, fibrin clots, formed in the presence of Aβ42 and without direct platelet involvement, were shown to be both structurally abnormal and resistant to degradation, potentially contributing to capillary stalling seen in AD and VaD [195, 276].

APP and platelets abnormalities in APP metabolism, APP secretases, ROS, and other intracellular signaling pathway biomarkers have been demonstrated in the platelets of AD patients [19]. Patients with AD display a modified pattern of APP forms, notably a diminished ratio (APPr) between the upper (120–130 kDa) and lower (106–110 kDa) immunoreactivity bands observed in platelets [277]. This altered pattern has not only been observed in patients diagnosed with AD, but also in the pre-dementia and preclinical stages [278, 279]. These changes are attributed to alterations in platelet APP metabolism, including reduced levels of platelet α-secretase (ADAM10) and its products, as well as enhanced platelet activity of β-secretase (BACE) and γ-secretase. Platelets derived from 31 patients with AD exhibited a marked alteration of APP, ADAM10 and BACE levels in the very early stages of the disease, where dementia can be barely inferred by neuropsychological assessments [277]. In addition, the number of platelet dense granules is decreased in AD patients [277]. These alterations suggest a shift to the amyloidogenic metabolism of platelet APP, leading to increased generation of Aβ peptides.

Mice models of AD exhibit notably elevated levels of plasma CLEC-2 in comparison to individuals with MCI which might be linked to elevated platelet activation in cognitive diseases [280]. Significantly higher baseline expression of both platelet activation biomarkers, activated glycoprotein IIb–IIIa complex and P-selectin, was observed in patients with AD with fast cognitive decline compared with AD patients with slow cognitive decline during a 1-year follow-up period [15]. Additionally, cerebral small-vessel disease patients, characterized by ischemic stroke and VD, exhibited transmural deposition of vWF, presumably originating from local endothelial synthesis [281]. In a Framingham Heart Study conducted on 1847 patients, platelet aggregation to adenosine diphosphate was shown to have a positive association with dementia risk [282]. A 15-year long population-based cohort study reported that higher vWF and low ADAMTS13 levels in plasma are associated with increased risk of dementia, again linking platelet activity to disease risk [283].

### Outlook

Extensive research has delved into platelet function in acute inflammatory processes, given their pivotal roles in clotting and healing post-injury [284]. However, their involvement in chronic inflammatory diseases like ADRD, where many of these mechanisms are dysregulated, remains poorly understood. Platelet receptor function and expression are profoundly altered in conditions such as AD, heightening predisposition to activation and aggregation. The heightened activation contributes to immune system dysregulation and systemic inflammation characteristic of these conditions. Very little is known about the underlying molecular mechanisms and physiological consequences of platelet-immune cell and platelet-vascular interactions. Also, the driver pushing platelets to increased reactive states still needs to be better understood. These are likely cardiovascular risk factors, environmental factors, and genetic predispositions. The diverse array of platelet receptors offers multiple avenues for novel treatment options. Investigations into blocking P2Y12, which regulates platelet activation and exocytosis, as well as PIEZO1, involved in sensing environmental alterations, represent promising therapeutic avenues under investigation. Additionally, changes in platelet granule content, including elevated levels Aβ and tau, can serve as early disease indicators and aid in quantifying disease progression. Given the relative accessibility of platelet and platelet-derived macrovesicles in the blood, they offer a promising avenue for developing cost-effective and readily accessible diagnostic tools. This underscores the importance of exploring platelet-related changes for enhanced disease detection and management.

## Electronic supplementary material

Below is the link to the electronic supplementary material.

Supplementary Material 1

## Data Availability

No datasets were generated for this study.
