# Supplementary material for: One immune cell to bind them all: platelet contribution to neurodegenerative disease
Source: Mol Neurodegener. 2024 Sep 27;19:65. doi: 10.1186/s13024-024-00754-4 (PMC11438031; doi:10.1186/s13024-024-00754-4)
Supplement: Supplementary file 1 — Supplementary Material 1 [file 13024_2024_754_MOESM1_ESM.pdf]

UNIVERSITY
OF MIAMI

Oliver Bracko, PhD
Assistant Professor
Cox Science Building
Department of Biology
Department of Neurology
University of Miami
Coral Gables, FL 33146

August/31/2024

Molecular Neurodegeneration Editorial Board

We are re-submitting our revised manuscript "**One Immune Cell to Bind Them All: Platelet Contribution to Neurodegenerative Disease".** We have recruited David Stegner, a world-leading expert in platelet biology, to the team to address some concerns from the reviewers. We have addressed all reviewer comments and suggestions, focusing on linking platelet function better to neurodegenerative disease throughout the manuscript and in the neurodegenerative disease section to align better with the journal's mission. Please find the submitted response to reviewers and the manuscript in a clean, revised, and revised version highlighting the changes we have made.

We thank you for taking the time to review and consider our manuscript.

Sincerely,

O.Bracko

Oliver Bracko

and the authors Gabriela Rodriguez Moore, Isabel Melo-Escobar, and David Stegner

UNIVERSITY OF MIAMI

Oliver Bracko, PhD
Assistant Professor
Cox Science Building
Department of Biology
Department of Neurology
University of Miami
Coral Gables, FL 33146

March/09/2024

Molecular Neurodegeneration Editorial Board

Dear Drs Vassar and Zheng,

We are submitting our manuscript "**One Immune Cell to Bind Them All: Platelet Contribution to Neurodegenerative Disease"** for consideration in the special section Glial and Vascular Contributions to Neurodegenerative Diseases in *Molecular Neurodegeneration*. In this manuscript, we highlight the pivotal role of blood platelets in chronic inflammatory disease, focusing on Alzheimer's disease and related dementias (ADRD). Several retrospective studies have shown that patients with elevated platelet receptor expression exhibit accelerated cognitive decline independent of traditional risk factors such as diabetes and hypertension. Additionally, platelets from AD patients exhibit heightened unstimulated activation compared to control groups. A handful of studies last year highlighted that platelet-derived factors, such as platelet factor 4, are functionally correlated with Alzheimer's disease progression in mouse models of AD. Furthermore, the pandemic has added a significant amount of data regarding platelet activation and its relation to long COVID and associated cognitive decline. Some of the concepts developed there could explain platelet changes seen in ADRD. Platelets have several diverse roles, including interacting with neutrophils, monocytes, T-cells, and endothelial cells. In addition, they can act as hubs sensing inflammation in the peripheral (Post-operative delirium, injuries, among many others) and neuroinflammation as seen in ADRD, triggering placket activation, which in turn drives further neurodegenerative processes leading to a vicious cycle.
In summary, recent findings across the field support the incorporation of platelets and their increasing importance in immune cell interactions and vascular inflammation in neurodegenerative diseases. As such, we are excited to share our manuscript, which discusses the divergent roles of platelets and highlights their connection to ADRD.

1. Schroer, A.B., Ventura, P.B., Sucharov, J. et al. Platelet factors attenuate inflammation and rescue cognition in ageing. Nature 620, 1071–1079 (2023). https://doi.org/10.1038/s41586-023-06436-3
2. Burkard P, Schonhart C, Vögtle T, et al. A key role for platelet GPVI in neutrophil recruitment, migration, and NETosis in the early stages of acute lung injury. Blood. 2023;142(17):1463-1477. doi:10.1182/blood.2023019940
3. Adair, B.D., Xiong, JP., Yeager, M. et al. Cryo-EM structures of full-length integrin αIIbβ3 in native lipids. Nat Commun 14, 4168 (2023). https://doi.org/10.1038/s41467-023-39763-0
4. Carlo Cervia-Hasler et al. , Persistent complement dysregulation with signs of thromboinflammation in active Long Covid.Science383,eadg7942(2024).DOI:10.1126/science.adg7942
5. Park, C., Hahn, O., Gupta, S. et al. Platelet factors are induced by longevity factor klotho and enhance cognition in young and aging mice. Nat Aging 3, 1067–1078 (2023). https://doi.org/10.1038/s43587-023-00468-0
6. Leiter, O., Brici, D., Fletcher, S.J. et al. Platelet-derived exerkine CXCL4/platelet factor 4 rejuvenates hippocampal neurogenesis and restores cognitive function in aged mice. Nat Commun 14, 4375 (2023). https://doi.org/10.1038/s41467-023-39873-9

UNIVERSITY
OF MIAMI

Oliver Bracko, PhD
Assistant Professor
Cox Science Building
Department of Biology
Department of Neurology
University of Miami
Coral Gables, FL 33146

We thank you for taking the time to review and consider our manuscript.

Sincerely,

Oliver Bracko

and the authors Gabriela Rodriguez Moore and Isabel Melo-Escobar

Reviewer suggestions:

1) Dr Tara Walker

The University of Queensland, Australia
Email: t.walker1@uq.edu.au

2) Dr Thierry Burnouf

College of Biomedical Engineering, Graduate Institute of Biomedical Materials and Tissue Engineering, Taipei Medical University, Taiwan
Email: thburnouf@gmail.com

3) Dr Ricardo Osorio

Department of Psychiatry at NYU Grossman School of Medicine, NY, USA
Email: Ricardo.Osorio@nyulangone.org

4) Dr Tony Wyss-Coray

Wu Tsai Neurosciences Institute, Stanford University, Stanford, CA, USA
Email: twc@stanford.edu
